# Against the Stream: religion and mental health – the case for the inclusion of religion and spirituality into psychiatric care

**DOI:** 10.1192/bjb.2017.13

**Published:** 2018-06

**Authors:** Simon Dein

**Affiliations:** 1 Queen Mary University of London, London, UK

## Abstract

This paper argues for the inclusion of religion and spirituality in psychiatric care. After discussing the antagonism of psychiatrists and psychologists to religion, I present a critical overview of studies examining the relationships between spirituality, religion and diverse aspects of mental health: depression, suicide, anxiety, delinquency, drug abuse and schizophrenia. The need to assesses the impact of religion in different faith groups is discussed. Measures of religious coping, both positive and negative, may provide a more accurate portrayal as to how individuals deploy religion in their lives than global measures such as belief and attendance. I highlight the fact that there is a dearth of research on ritual, prayer and other aspects of religious experience. While many studies demonstrate positive effects of religion on mental health, others find detrimental effects. Finally I examine the clinical implications of these findings.

**Declaration of interest:**

None.

Psychiatry and religion have traditionally had a difficult relationship. The views of Freud and others such as Albert Ellis have negatively affected the attitudes of mental health professionals pertaining to the mental health effects of religion. Religious beliefs and practices are widely seen to be ‘primitive’, dependency forming, guilt inducing, non-empirical and necessarily bad for mental health. However, compared with psychologists and psychiatrists, the patients consulting them have been found to have higher levels of religiosity – there is a so called ‘religiosity gap’ between mental health professionals and those they treat.^1^ For many people, religion is not only important in their lives but the central aspect of coping with life stresses. There is, moreover, evidence that psychiatrists tend to ignore religion; it is rarely part of standard psychiatric assessment and treatment. As Rosmarin *et al* state^2^:

[R]eligious beliefs are often ignored in the context of treatment as mental health professionals are often ill-trained in the assessment of these factors in clinical settings. This deficit creates a reticence to broach this topic in psychiatric research and practice_,_ which in turn perpetuates assumptions throughout the field that these facets are tangential to human functioning and a side issue in treatment. Protocols for assessment seem to ignore religious beliefs and there seem to be few interventions that take account of religious and spiritual beliefs.

Here I argue against the assumption that religious beliefs are largely irrelevant to clinical psychiatric practice.

## Recent findings

In the past 20 years, there has been escalating research focusing on the relationships between various dimensions of religiosity and mental health. To date, several thousand studies demonstrate positive associations between the two.^3^^,^^4^ Results indicate that those who are more religious generally fare better in terms of mental health.

The presence of religious faith is associated with greater hope, increased sense of meaning in life, higher self-esteem, optimism and life satisfaction. In terms of depression, Koenig (2012) reports that of 70 prospective cohort studies, 39 (56%) indicated that greater religion/spirituality (R/S) predicted lower levels of depression or faster remission of depression, seven (10%) predicted worse future depression and seven (10%) reported mixed results (both significant positive and negative associations depending on R/S characteristics).^5^ Higher religiosity has also been associated with lower rates of suicide,^6^ reduced prevalence of drug and alcohol misuse,^7^ and reduced delinquency.^8^ Findings in relation to anxiety are rather mixed. Although some studies demonstrate reduced anxiety rates, others indicate that anxiety levels are increased in the more religious.^9^ There are few studies relating schizophrenia to R/S. Recent studies from Switzerland suggest that religious individuals with psychotic illnesses frequently pray and read the Bible to facilitate coping with their voices, and that higher levels of religiosity may increase medication adherence.^10^ Little work has been conducted on explanatory models, treatment-seeking and outcomes in this condition.

Although the focus of the existing literature on religion and mental health predominantly relates to Christianity, there has been recent work on Islam,^11^ Judaism^12^ and Hinduism,^13^ similarly suggesting that those who are religious have better indices of mental health. Furthermore, these studies suggest that religious beliefs have different effects on mental health depending upon the faith group of subjects.

Global measures of religion such as belief may reflect dispositional religiousness rather than how people actually deploy religion during crises. As Pargament and colleagues^14^ (p. 521) state, ‘It is not enough to know that the individual prays, attends church, or watches religious television. Measures of religious coping should specify how the individual is making use of religion to understand and deal with stressors.’ There is evidence that some forms of religious coping are protective in the wake of adverse life events, while others may be maladaptive. This author contends that there are two sorts of coping: positive religious coping and negative religious coping.^15^ The former (e.g. benevolent religious appraisals, religious forgiveness) reflects a secure relationship with God and generally results in improved mental health. By contrast, the latter (e.g. reappraisals of God's powers, feeling abandoned or punished by God) reflects a weak relationship with God and is associated with worse mental health indices. There is some recent discussion of the psychological implications of theodicy – the defence of God's goodness and omnipotence in view of the existence of evil.^16^

There a dearth of research examining the mental health effects of ritual, prayer and other aspects of religious experience. Although popularised in William James’ classic *The Varieties of Religious Experience*,^17^ religious experience has attracted less research than attendance, beliefs and coping, possibly because of its subjective nature and lack of clarity in definition. The focus has been on three main areas: mysticism, conversion and religious hallucinations. Religious conversion has generally been found to enhance mental health. There are phenomenological parallels between mystical and psychotic states (including visions, voices, loss of sense of self) although the outcomes are different. While mystical experiences typically affect mental health positively, psychosis is generally a negative experience.^18^ There has been some phenomenological research on hearing God's voice among Pentecostal Christians in London. Among this group, hearing his voice is normative and many reported its utility in resolving distress.^19^ Finally, one study examined the differences between prophecy and loss of agency and thought insertion in schizophrenia. In contrast to schizophrenia, in prophetic experiences agency is preserved.^20^

However, religion may also have a negative effect on health through inducing guilt and dependency, and in extreme cases may precipitate suicide (e.g. in extreme cultic groups).^21^ Of great contemporary interest, the wider social impact of mental health on radicalisation remains to be investigated. Bhui has provided initial data suggesting that among Pakistani and Bangladeshi Muslims, those endorsing the most sympathy for violent protest and terrorism were more likely to report depression.^22^

## Criticisms

There have been a number of criticisms of the above findings.^23^ First, there may be selection biases in recruiting subjects. Second, more work needs to be conducted on the non-religious and their mental health associations, including atheism and agnosticism.^24^ Third, the vast majority of these studies have focused on religious attendance and beliefs among North American Christians, and findings cannot be generalised to other religious groups. Fourth, some people are spiritual – connected to a higher power from which they derive meaning – although not belonging to and participating in institutionalised religion. The similarities and differences between religion and spirituality warrant further research, as do the associations of spirituality with mental health. Finally, measurement scales need to be more culturally and theologically sensitive.^25^

## Clinical implications

Given the above findings, what are the clinical implications? It is clear that the assessment of religious belief should be included routinely in psychiatric assessment. It may be that the incorporation of religious activities such as prayer, Bible reading and ritual into cognitive–behavioural therapy (CBT) could enhance its effectiveness. Evidence suggests that Christian-based CBT is more effective among Christian patients with depression and anxiety than traditional non-religious CBT.^26^ Future work in this area should concentrate on which therapies are efficacious, for which patients, and which therapists should be conducting them. Pargament provides a number of illustrative examples of how spirituality can be incorporated into psychotherapy.^27^

## Conclusion

There is now a voluminous literature examining the relationship between religion and mental health. On balance, it appears that being religious enhances mental health. Future work in this area needs to explore the clinical implications of these findings, and how working with patients’ theological constructs such as guilt, sin and forgiveness helps to promote recovery. Most importantly, both clinical work and research need to be more sensitive to cultural and theological issues.^28^ The Royal College of Psychiatrists^29^ and the WPA^30^ have published two Position Statements on spirituality, religion and clinical care.

## About the author

**Simon Dein**, Honorary Professor at Queen Mary University of London and at Durham University, Honorary Senior Lecturer at University College London. He is on the Executive Committee of the Spirituality and Psychiatry SIG.
